# Supplementary material for: Tailoring electron beams with high-frequency self-assembled magnetic charged particle micro optics
Source: Nat Commun. 2022 Jun 9;13:3220. doi: 10.1038/s41467-022-30703-y (PMC9184583; doi:10.1038/s41467-022-30703-y)
Supplement: Supplementary file 1 — Supplementary Information [file 41467_2022_30703_MOESM1_ESM.pdf]

Supplementary information

Tailoring electron beams with high-frequency self-assembled magnetic charged particle micro optics

R. Huber[1,2+], F. Kern[3+], D. D. Karnaushenko[1,4], E. Eisner[1], P. Lepucki[3], A. Thampi[3], A. Mirhajivarzaneh[1], C. Becker[1,4], T. Kang[1], S. Baunack[1], B. Büchner[3,6], D. Karnaushenko[1,4*], O. G. Schmidt[1,2,4,5*], A. Lubk[3,6*]

[1]*Institute for Integrative Nanosciences, Leibniz IFW Dresden, 01069 Dresden, Germany.*

[2]*Material Systems for Nanoelectronics, Chemnitz University of Technology, 09107, Chemnitz, Germany.*

[3]*Institute for Solid State and Materials Research, IFW Dresden, Helmholtzstraße 20, 01069 Dresden, Germany.*

[4]*Center for Materials, Architectures and Integration of Nanomembranes (MAIN), Chemnitz University of Technology, 09126, Chemnitz, Germany.*

[5]*Nanophysics, Faculty of Physics, TU Dresden, 01062 Dresden, Germany.*

[6] *Institute for Solid State and Materials Physics, TU Dresden, Germany*

[*] Correspondence and requests for materials should be addressed to A.L. (email: a.lubk@ifw-dresden.de), or to O.G.S. (email: oliver.schmidt@main.tu-chemnitz.de), or to D.K. (email: d.karnaushenko@ifw-dresden.de).

[+] R.H. and F.K. contributed equally to this work

Supplementary Note 1 | Fabrication details

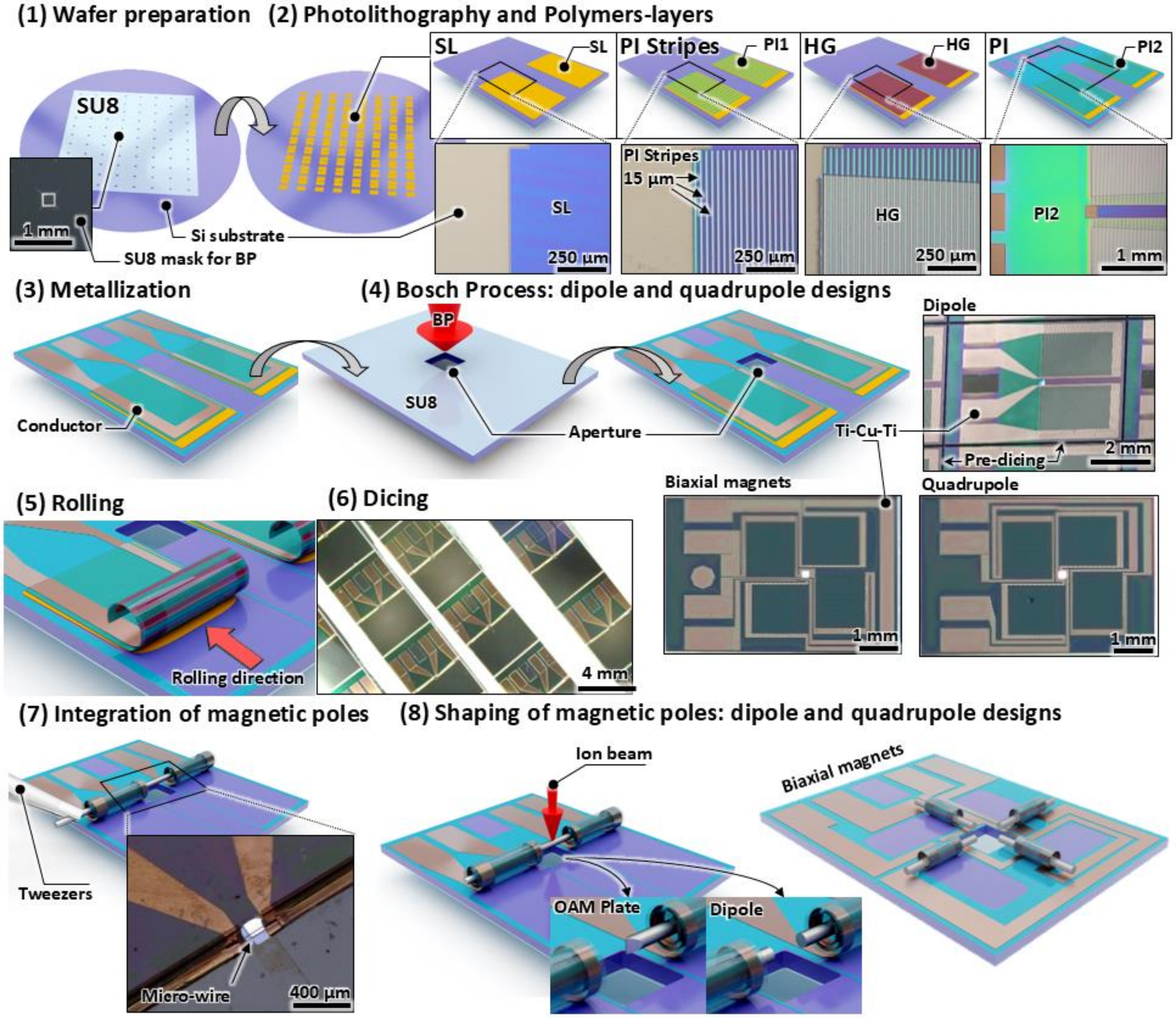

Supplementary Figure 1 | Manufacturing steps of the µMCPO. (1), in the first step a mask is photo patterned in the backside of the silicon wafer, the inset shows one 200x200 μm^2 hole in the SU8 layer. (**2**), on the top side of the wafer the polymeric technology is applied, the inset shows the subsequent steps: first sacrificial layer (SL) is applied, then the polyimide stripes (PI1), then the Hydrogel (HG) and finally the last polyimide layer (PI2), microscopy close-ups are provided for all steps. **(3)**, the conducting layer is patterned (Ti-Cu-Ti). **(4),** the wafer is flipped in order to apply the Bosch process from the backside, producing the aperture in the middle. The four pole elements have two designs for the electrical interconnects, the biaxial magnets and the quadrupole as shown in the micrographs. The 6-inch wafer is pre-diced prior to the rolling process **(5)**, in which the SL (yellow) is etched away and the tube self assembles. Once the tubes are ready the wafer is manually diced **(6)**, into single samples and the magnetic core is inserted **(7)** inside of the polymeric tubes as shown in the microscope image where one micro-wire inserted in both tubes. **(8)**, The sample is now ready for the customization of the micro tip by FIB milling for either monopole, dipole, biaxial magnets configuration or quadrupole configuration. The latter two employ the same flat tip geometry.

Design: The sample design considers both the final goal geometry and the fabrication requirements. In particular, the footprint of the µMCPO device is limited by the sample holder, a Protochips Fusion™ TEM, at 4x6 mm^2. In this area, all the polymeric layers and interconnects need to be photo patterned. A further challenge is the integration of the Bosch etching process (BP), which must be applied from the backside of the wafer. This work made use of a variety of wafers, as shown in Supplementary Table 1.

Diameter [Inch]	Thickness [µm]	Doping	Conductivity [Ohm·cm]	Type	Polished
3	200 ± 25	Boron	0.009 – 0.01	Cz (100)	Both sides
6	425 ± 15	Boron	0.01 – 0.02	Cz (100)	Both sides
6	675	Boron		Cz (100)	One side

Supplementary Table 1: wafers that are used for prototyping. A number of wafers are employed during this work, they vary in thickness diameter and conductivity, best results were achieved with the 3-inch wafer for prototyping and with the 6-inch wafer double side polish.

To achieve directional self-assembled rolling of the tube, the rolling process must be tweaked. The tube is anchored on one side with width w_r of the polymeric layer that extends for a length l_c, thus the aspect ratio of the planar surface can be defined as follows: $r_c = \frac{l_c}{w_c}$ the length along the rolling direction over the length perpendicular to the rolling direction, see Fig. 2 a. This means that for a given w_r, the higher the aspect ratio, the higher the number of windings the tube can have. D. D. Karnaushenko employs anchors to direct the self-assembled rolling[1]. This method allows to roll surfaces with high aspect ratios, in his case, up to $r_c = 3.13$. C. Becker[2] employs a trapezoidal shape of the swelling layer. This approach works for aspect ratios below one, in his case $r_c = 0.67$. In this work, the planar structure has a length of 2.6 mm along the rolling direction and a width of 1.775mm, this means an aspect ratio of 1.48, which is bigger than one and thus dictates an anchor approach. However, the anchors would add an extra footprint on the sides of the tube, which would interfere negatively with the electron beam. Therefore, an alternative approach that takes inspiration from P. Cendula [3], where high aspect ratio tubes are achieved by wrinkling of the membrane itself. In this work, rigid stripes are added underneath the swelling layer in order to add one-dimensional rigidity and thus direct the rolling direction, as seen in Fig. 2 a and b.

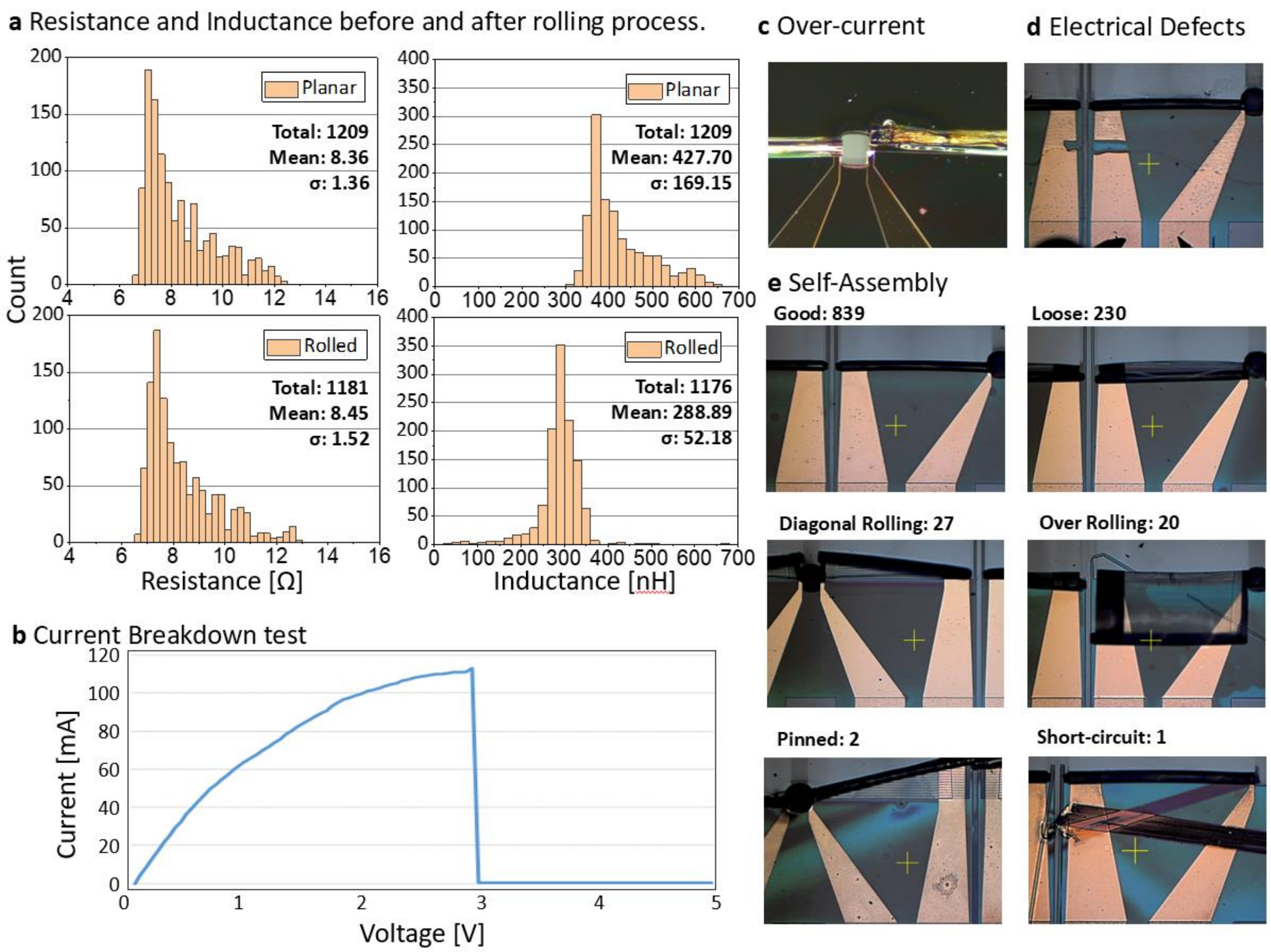

Supplementary Figure 2 | Yield and electromagnetic parameters distribution. a, The distribution of the electrical resistance and of the inductance of every sample on a 6-inch wafer before and after the self-assembly process. **b**, Typical behavior of the coil current for an increasing voltage over time, this particular coil breaks with a current close to 110 mA. **c,** When the current is too high, the joule effect heats up the coil, which in return burns the polymeric tube. **d,** a scratch on a sample ruins the electrical contact of one sample. **e,** After the self-assembly rolling process four main categories can be distinguished: 839 tubes are well assembled, 230 tube are a bit loose (these tubes can be employed and fixed when the micro-wire is inserted), 27 tubes rolled in a diagonal direction (2 were pinned down from one side) and 20 tubes did not roll up properly. There is also a short-circuited sample caused by a loose copper foil from the lift-off process.

First Step - Silicon Wafer preparation: The BP requires a mask that cannot be applied on top of the polymer layers without damaging them during removal; additionally, the polymer layers must be applied on the wafer prior to the BP, because liquid polymers are challenging to coat on a wafer with holes. As a result, the mask is applied to the backside of the wafers and does not need to be removed after the BP, leaving the top surface free for the polymer layers. This, however, requires aligning the structures on both sides of one wafer with each other, which was accomplished using an MA6 Mask

Aligner (SÜSS MicroTec SE, Garching, Germany) mask aligner that has the back-side alignment technique option. Here, as the mask, we used a standard photo-patterned SU8-10 layer: The wafers were first dried on a 120°C hotplate. After cooling, the wafers were inserted in an SPIN 150i (SPS-Europe) spincoater and Ti-prime was coated for 20 seconds at 2000 rpm, followed by 2 minutes at 120°C on a hotplate. After cooling down to room temperature, the SU8-10 was coated in a two-step program, 500 rpm for 10 seconds and then 1500 RPM for 30 seconds. Followed a two-step baking process, the wafers were placed on a hotplate and backed at 65°C for 2 minutes and then at 95°C for 5 minutes. Following that, the areas that were supposed to stay on the wafer were exposed with a dose of 30 mW/cm^2 for 1.5 seconds in the mask aligner. It followed a second two-step baking process: 65°C for 2 minutes and then 95°C for 3 minutes. The wafers were then developed by immersion in acetate (MR DEV 600) for 4 minutes with soft agitation. After that, the wafers were rinsed with isopropanol and dried with a pressured stream of nitrogen. Lastly, with the SU8 layer facing down, the wafers were placed on a hotplate at 220°C for 5 minutes. Supplementary Figure 1 (1) shows the schematics of the backside of one wafer with an SU8 layer and its micrograph shows one hole in the SU8 layer, which is around 20 µm deep and has an area of 200x200 μm^2 (this is a dipole design, the quadrupole design has a smaller aperture area of 150x150 μm^2). To electrically insulate the other side of the wafers, an Al_2O_3 layer was applied on the empty side of the wafers with a FlexAL (Oxford Instruments Plasma Technology) atomic layer deposition system (ALD). After loading the wafer into the ALD chamber, it ran the rinse and pump cleaning program followed by 400 ALD steps at a chamber temperature of 220°C. The thickness of the deposited layer was estimated to be around 40 nm. The last step in the wafer preparation is the silanization process. The wafer was cleaned with acetone, isopropanol. Then they were cleaned for 15 minutes at 600W with O_2 flow of 200 sccm in a GIGAbatch 310M (PVA Metrology & Plasma Solutions GmbH, Wettenberg, Germany) O_2 plasma system. After that, the wafers were dipped into water and dried with a N_2 pressurize stream. Finally, the wafers were inserted in a vacuum oven with 200 µL 3-Methacrylpropyltrimethoxysilane in a glass dish and the temperature of the oven was set to ramp up to 150°C and down to room temperature.

Second Step - Photolithography, Polymer technology and Metal layer: In the fabrication procedure, four polymeric layers were employed. In this order, a sacrificial-, rigid-, swelling-, and rigid-layer are photo-patterned (resp. SL, PI1, HG and PI2), as shown in Supplementary Figure 1 (2). The synthesis of the polymer layers follows the process described in previous works [4]. The patterning of the polymers requires 5 steps for one wafer: The chosen polymer is (1) coated with an optimal rpm

in a spincoater, and afterwards the wafer is (2) soft baked on a hotplate at a polymer dependent temperature and duration. Now the polymer-layer is ready for the (3) exposure step mask aligner, the structures are aligned, either to each other or to the SU8-10 layer on the backside of the wafer using the back-side alignment feature. Following the (4) development of the polymer by immersion, the solution and its duration depend on the material. The wafer is then rinsed with acetate MR Dev 600 for 10 seconds and dried with a N_2 pressurized stream. Next, the wafer is (5) hard baked at 220°C on a hotplate. The previous steps were repeated for all the needed polymer-layers, n.b. the rpm value may need optimization to reach the desired thickness of the applied layer. Supplementary Table 2 provides the variables employed for the various materials.

	(1) Spin coating	(2) Soft bake		(3) Exposure	(4) Development		(5) Hard bake	Thickness
	rpm	[°C]	[min]	[mJ/cm^2]	Solvent	[MM:SS]	[min]	[nm]
SL	1500	35	10	280	DI water	00:30	10	300 ± 100
PI1	3000	50	5	600	NEP Sol	04:00	5	600 ± 100
HG	4000	40	5	450	DEGMEE	01:30	10	600 ± 100
PI2	3000	50	5	660	NEP Sol	03:00	5	600 ± 100

Supplementary Table 2: The photolithography parameters. The applied polymers layers are sacrificial layer SL, the polyimide stripes PI1, hydrogel HG, and a last polyimide layer PI2. The solvents are: DI water (deionized water), DEGMEE (Diethylenglykol-monoethylether), or NEP Sol (NEP(1-Ethyl-2-pyrrolidon)-DEGMEE-ethanol in 4-2-1 volumetric parts).

Third Step – Metallization: The next step is the metal deposition and patterning that forms the electrical contacts on top of the polymer layers on the wafers, as shown in Supplementary Figure 1 (3). For this purpose, the lift-off technique was used with AZ 5214E (MicroChemicals) image reversal photoresist as a sacrificial layer. The wafers were coated with the photoresist at 3500 rpm for 30 seconds in a spincoater. They were then placed on a hotplate at 90°C for 5 minutes before being loaded into the mask aligner for alignment and exposure with 45 mJ/cm^2 power. Afterwards, the wafers were placed on a hotplate at 120°C for 2 minutes, before being flood exposed with a 300 mJ/cm^2 dose. The photoresist was developed by immersion in AZ 726 MIF, a TMAH based solution, and then it was rinsed with deionized water. The wafers are now inserted into a sputtering device (HZM-4P, Von Ardenne) with a titanium and a copper target. Sputtered is first a 5 nm ± 2 nm titanium adhesion layer, then a 300 nm ± 100 nm copper layer, and finally a 5 nm ± 2 nm titanium protective layer. The AZ 5214E sacrificial layer was then removed by immersing the wafer in acetone, then rinsing it with isopropanol and drying with an air gun.

In preparation for the following stage, the BP, a protective layer was applied on top of the structures: the wafers were coated with AZ 520 D (MicroChemicals) protective coating at 2000 rpm for 30 seconds, followed by 10 minutes on a hotplate at 90°C.

Fourth Step - Bosch Process*:* Because of their compatibility with the BP, which has been established at an industrial level for silicon etching, Si wafers were chosen as the principal substrate. The BP was used to etch the aperture through which the electron beam traverses the µMCPO element, see Supplementary Figure 1 (4). Although 3-inch wafers were the preferred choice for prototyping, the BP technique was demonstrated on a 6-inch wafer. The BP was carried out with some adjustments in a PlasmaPro 100 ICP (Oxford Instruments Plasma Technology) reactive ion etching system (RIE). The Si-wafer with the SU8-10 mask pointing upwards is secured with polyimide tape on the aluminium carrier of the RIE (the tape also covers the Si exposed areas on the wafer edges). The carrier is loaded first in the pre chamber, then in the main chamber. Before and after the BP program, a cleaning process of the chamber consisting of sequential pumps and purges with argon is applied. Typically, the BP consists of two major steps: passivation and etching, which alternate in a continuous cycle. However, a third stage to stabilize the plasma is introduced to the cycle as follows: passivation, etching and transition. The parameters employed for the BP cycles are shown in Supplementary Table 3. The etching rate is estimated to be 0.7 µm for cycle, or ((0.7/(7+7+3))*60) 2.47 µm/min. Even though a stopping layer for the BP is not required, the Al_2O_3 layer serves as one because the etching rate of the BP is drastically lowered on it. This implies that an Al_2O_3 membrane may persist at the bottom of the hole. However, this membrane breaks when the AZ 520 D resist is removed because of the volumetric stress created by the swelling resist.

	Duration [s]	Pressure [mBar]	RF Power [W]	ICP Power [W]	SF6 [sccm]	C4F8 [sccm]	Temperature [°C]
Passivation	7	0.04	0	500	0	95	20
Etching	7	0.03	10		85	0	
Transition	3	0.03	0		65	30	

Supplementary Table 3: The Bosch process is performed in a PlasmaPro 100 ICP (Oxford Instruments Plasma Technology) at a stable temperature of 20°C and a constant ICP Power set at 500 W. The RF power is switched on only during the Etching step. The BP starts with the Passivation program, follows the Etching program and then there is the Transition program before the cycle starts again with the Passivation program.

Fifth Step - Rolling and Assembly: After the BP, the wafers were loaded into a SS10 (ACCRETECH (Europe) GmbH) blade dicer. The cut depth was set to make a 100 µm deep cut, allowing all the

samples on one wafer to be rolled in parallel and diced at a later stage. The protective coating layer was removed with acetone, rinsed with isopropanol and dried with an air gun. Supplementary Figure 1 (5) depicts the rolling process of a single sample when the wafer is placed inside of a beaker with an etching solution that removes the polymeric sacrificial layer. The etching solution was made starting with a 1:30 ratio by mass of EDTA (Ethylen Diamine Tetra Acetic acid, $[CH_2N(CH_2CO_2H)_2]_2$) and H_2O, this acidic solution is adjusted with NaOH pellets until the solution reaches a 6.9 Ph, Benzotriazole (C6H5N3) is added in a 1:10 ratio by mass to EDTA and finally ethanol is added in a 1:9 ratio by volume to H_2O. After the sacrificial layer is removed, the Ph of the solution is increased by adding 2% NaOH solution. The Ph is raised up to around 10. The Ph of the solution influences how tight the tubes are. For instance, the final diameter of the tubes varies from 80 to 160 µm. The rolling process is enabled by volumetric stress between the polyimide layer and the hydrogel, see Fig.2 b. At this stage, the wafer or sample is removed from the etching solution and it is rinsed by immersion in a solution of H_2O and ethanol in a 4:1 ratio by volume. The sample or wafer is then placed on a lab paper towel to dry.

Sixth Step – Dicing: The 6 inch wafer undergoes a pre-dicing process in the SS10 (ACCRETECH (Europe) GmbH) blade dicer device after the Bosch Process step. At this stage of the fabrication, all the rolled samples are still attached together. However, thanks to the pre-dicing, it is possible to manually break the wafer into stripes, see Supplementary Figure 1 (6), and subsequently into single samples.

Moreover, it is important to note that it is also possible to set the cut depth of the blade dicer to cut through the whole wafer before the rolling process takes place. In this latter case, the rolling process can be down scaled to single samples if desired. This is advantageous for tuning the rolling process. This approach was employed mostly on the 3-inch wafers.

Seventh Step – Integration of magnetic poles: Every single sample is elaborated individually from this point forward. Following the dicing of the wafers into single samples, samples capable of withstanding at least 100 mA (if not 150 mA) were preselected for the final fabrication steps. A few pieces of CoFeSiB soft magnetic micro-wires with a length of about 2 mm were cut with scissors. Following that, the micro-wire piece was inserted inside of the aligned tubes on the sample with the help of tweezers, as shown in Supplementary Figure 1 (7). The micrograph shows a 10 µm diameter wire inserted through the tubes of a dipole; for an SEM image, see also Supplementary Figure 3 a. To facilitate this step, dipoles and quadrupoles were previously immersed in a water base solution. This improved the tweezers' control over the micro-wire. After the micro-wire was inserted, the

samples were removed from the water solution and allowed dry. When necessary, the cores were glued inside of the tubes, as shown in Supplementary Figure 3 e. Lastly, the samples were cured in a fume hood for at least 3 hours on a hotplate at 70°C, which helps to dry the solvents of the glue.

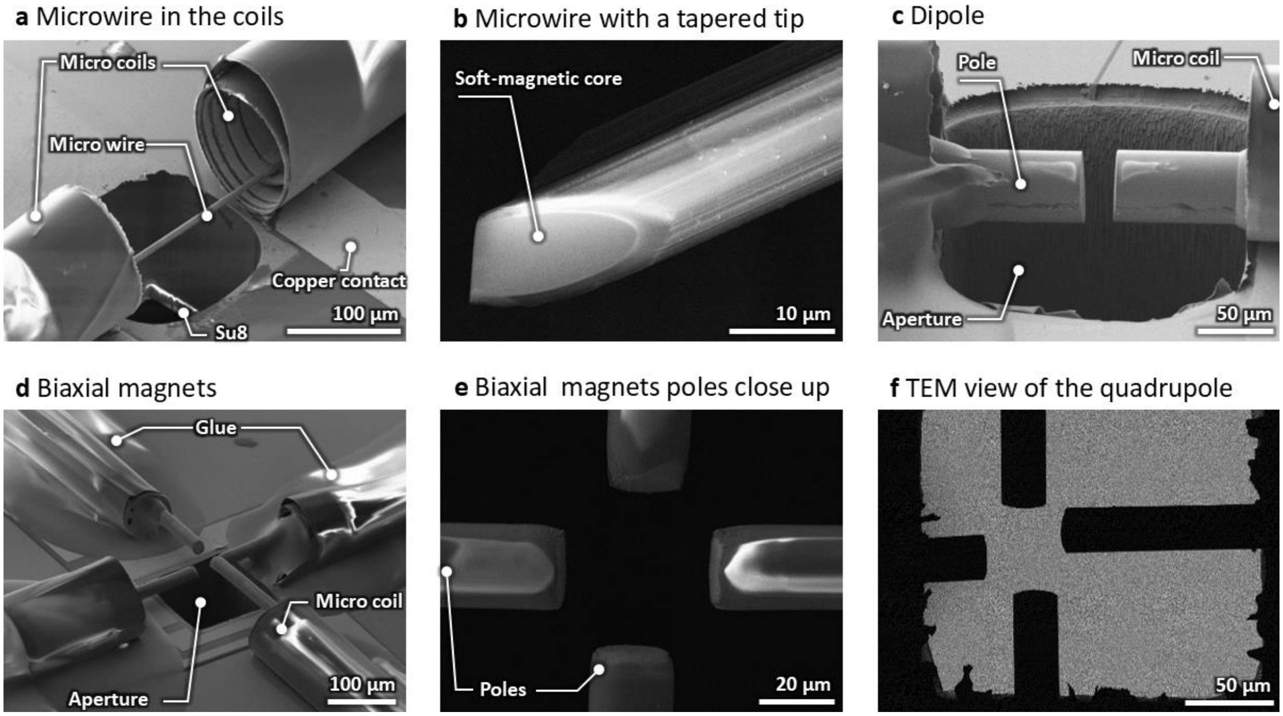

Supplementary Figure 3 | Custom tip geometries. a-e Scanning electron microscopy (SEM) images of the micro-poles inserted in the micro-coils. The SEM was operated at 5 kV acceleration voltage employing a secondary electron detector for all images but **b**, for which 15kV and "in lens" electron detector was used. **a,** A 10 µm micro-wire inserted in a µMCPO ready for the FIB milling process. **b**, Close up of the tapered tip for the vortex plate, here the coated glass is distinguishable from the soft magnetic material. **c,** Dipole with 50 µm thick wires and a narrow gap. **d**, Biaxial magnets during milling process. **e**, Close-up of the finished biaxial magnets, the micro-wire is 20 µm thick. **f**, STEM overview image of a quadrupole, the poles are not perfectly aligned.

Eighth Step – Shaping of the magnetic poles: The micro-wire tip can be shaped to the desired geometry by a focused ion beam (FIB). The FIB milling process took place on the poles, where different geometries were formed. For the vortex phase plate, the microwire was shaped into a tapered tip (see Supplementary Fig 3 b), while the other side of the micro wire was cut next to the residual coil. This type of sample was also converted into a dipole geometry, as shown in Supplementary Fig 3 d, where the used wire is 50 µm thick, but other thicknesses can also be employed, such as in Fig f. Supplementary Fig 3 e depicts a quadrupole during the FIB process and

f depicts a close-up of the finished quadrupole. This quadrupole is made with 20 µm thick micro-wires. After the FIB step, the sample is ready to be loaded into the TEM.

Design of the quadrupole and of the biaxial magnets: To fabricate quadrupole µMCPO capable of modulating an electron beam in 2D, a new design is required where four micro-electromagnets are arranged around the aperture. All the previous fabrication steps are applied to a new design, but because the number of contacts to the outside is limited to four, each coil is wired in series with the coil on the opposite side of the aperture. Because this configuration doesn't allow to drive individually, we didn't build a µMCPO that could be both a focusing quadrupole and a deflecting biaxial magnet µMCPO device. This is why we created two metallization designs to meet the needs of both applications, as shown in Supplementary Figure 1 (4). The tips for the elements in both biaxial magnets configuration and quadrupole configuration are shaped in a flat way similar to the dipole (see Supplementary Figure 1 (8)).

Supplementary Note 2 | Self-Assembly yield.

One 6-inch wafer was thoroughly examined to test the quality of the self-assembled process. Supplementary Figure 2 a depicts the histograms of the resistance and inductance distribution before and after the 3D self-assembly rolling process. In the planar state the yield is nearly perfect; out 1209 tubes on the wafer, only one that has a scratch is defective (see Supplementary Figure 2 d). The rolling process had some effects on the electrical resistance, which increased marginally from 8.36 Ω ± 1.36 Ω to 8.45 Ω ± 1.52 Ω, as did the standard deviation, however, the rolling process did damage 28 samples. The inductance, on the other hand decreased, from 427.70 nH ± 169.15 nH to 288.89 nH ± 52.18 nH. This phenomenon can be explained by the smaller loop area from a planar point of view as discussed in [1]. In this case 33 samples were considered to be damaged by the rolling process. The electrical study shows the consistency of the fabrication and rolling process, however, a visual test discerned other defects among the rolled structures. Supplementary Figure 2 e, shows different outcomes of the self-assembled coils. There were 839 coils that were considered well assembled, there were 230 coils that were a little loose; however, this coils can still be used in the next fabrication steps, where the tightness of the tube can be tuned. The following tubes were deemed defective. There were 27 tubes that rolled in a diagonal direction, there were 20 tubes that did not roll properly or didn't finish the rolling process. Two tubes were pinned down to the wafer and one tube was shorted by a remaining copper foil from the lift-off process.

Supplementary Note 3 | Differential Phase Contrast Measurement

The magnetic fields in the devices in this paper were reconstructed by means of a center of mass differential phase contrast (COM DPC) measurement in a double-corrected FEI Titan[3] 80-300 TEM. In this setup, a focused electron beam is scanned over the sample. For every scanning position, the corresponding diffraction disk is recorded in the far field of the object plane. The projected transverse magnetic stray field of the sample (of effective thickness *t)* **B***t* was reconstructed by evaluating the measured shift of the center of mass of the diffraction disk **d** in terms of the projected magnetic field:

$$\mathbf{d} = \frac{e\mathbf{B}t}{p_z}L \quad (1)$$

Here, e is the elementary charge, p_z the kinetic momentum of the beam electron and L the effective electron optical distance or camera length between the object and the CCD detector. For this work a special low magnification DPC mode was established, in which an excitation of the objective lens of just 4% enabled large camera lengths to record the deflection with a very high precision. The resulting camera length was calibrated by electron diffraction on a fine structured cross grating (2160 l/mm) to 13.16 m.

Finally, the corresponding phase of the electron wave $\varphi(\boldsymbol{r})$ exiting the sample may be derived by numerically integrating the local variations of the projected magnetic field (see Fig 3c):

$$\frac{\hbar}{e}\partial_x\varphi(r) = B_y t;\ \frac{\hbar}{e}\partial_y\varphi(r) = -B_x t \quad (2)$$

Supplementary Note 4 | Vortex beam generators

The „vortex“ beams generated by the single pole µMCPO device in this paper exhibit a significant deviation from an ideal circular ring shape, i.e., only a half ring is visible in the far field of the single pole µMCPO device. A previously reported vortex beam generator based on a ferromagnetic nanowire (see Béché et al.[5], Fig. 3 and Suppl. Fig. 2) suffered from similar imperfections and we attribute them to two main causes: (I) Charging of the microwire (including the glass coating) in the electron beam and (II) magnetic stray fields emerging from the sides of the wire (in particular at the tapered regions). While the first effect produces a marked asymmetry of the vortex (see, e.g., Pozzi et al.[6]), the second restricts the azimuthal range of the deflection and hence the arc angle in the far field. We studied (and tried to improve) these aspects by FIB preparing a lamella-like tip (in order

to reduce field lines emerging from the sides, see Supplementary Figure 4) and by coating a tip with Au (not shown). Both measures improved the vortex shape. Note, however, that significant deviations from a real vortex persist, presumably due to the remaining magnetic field lines emerging from the sides of a non-homogeneously magnetized poles (indeed, our vortex state microwires can sustain complicated tilted vortex or flux closure domains in the tip region).

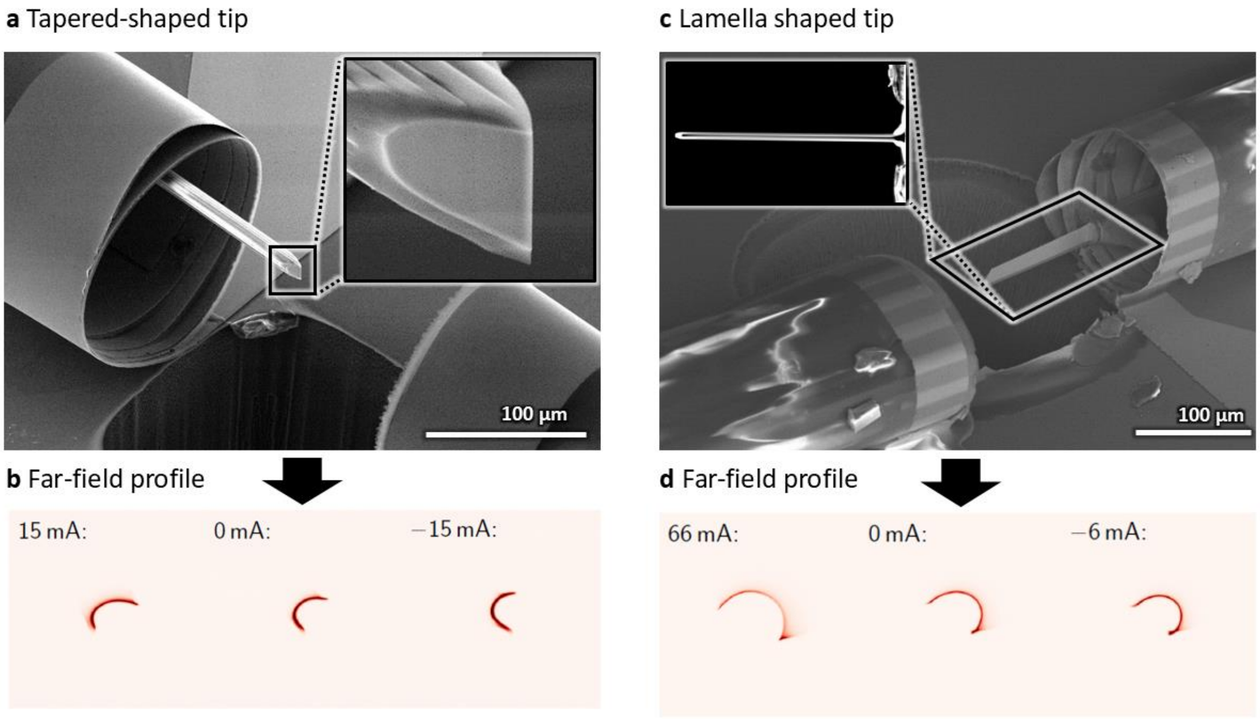

Supplementary Figure 4 | Vortex beam generators utilizing differently shaped microwires. a, c show FIB fabricated microwire tip of triangular tapered and lamella shape. **b**, **d** shows the corresponding electron beam in the far field of the device exhibiting half vortex character with a larger azimuthal range in case of the lamella-shaped tip (reducing the magnetic stray fields emerging from the sides of the wire).

Supplementary Note 5 | Quadrupole lenses

We evaluate the focal length of the miniaturized quadrupoles by means of geometrical optical considerations, employing the intercept theorem. In the object plane, where the quadrupole is located in this measurement, the diameter of the round parallel illumination was 37.8 µm, as determined in the TEM image with a gold cross grating (2160 l/mm) as reference. The µMCPO element in a quadrupole configuration at I_c = 100 mA focused the beam anisotropically in an elliptical diffraction disk in the far field with the semiminor axes of this elliptical beam measuring 1371 µm and 1061 µm on the detector. Following the intercept theorem, a focal length of 46 mm

± 8 mm measured at an effective camera length of 1.4 m and an acceleration voltage of 300 kV. We calibrated the camera length with the systematic reflections of a gold sample.

Supplementary Note 6 | Deflection analysis

In order to analyze the achievable deflection power of the biaxial µMCPO as well as the achievable shift in the specimen position, the µMCPO was placed in C3 aperture position inside of the double-corrected FEI Titan[3] 80-300 TEM. To facilitate HF signal supply for the device, we modified a custom-made electrical biasing aperture holder based on the NanoEx i/v specimen holder provided by ThermoFisher (FEI). The deflection properties of the beam were investigated by applying a harmonic signal of varying frequency (from 16 kHz to 500 MHz) to the coils in *x*- and *y*-direction. Consequently, the electron beam would follow a harmonic oscillation if the produced magnetic fields are uniform and free from hysteresis effects, regardless of the oscillation frequency. To test that hypothesis and to characterize deviations from that ideal behavior we collected the integrated intensity of the deflected beam in the far field of the deflecting microcoil device.

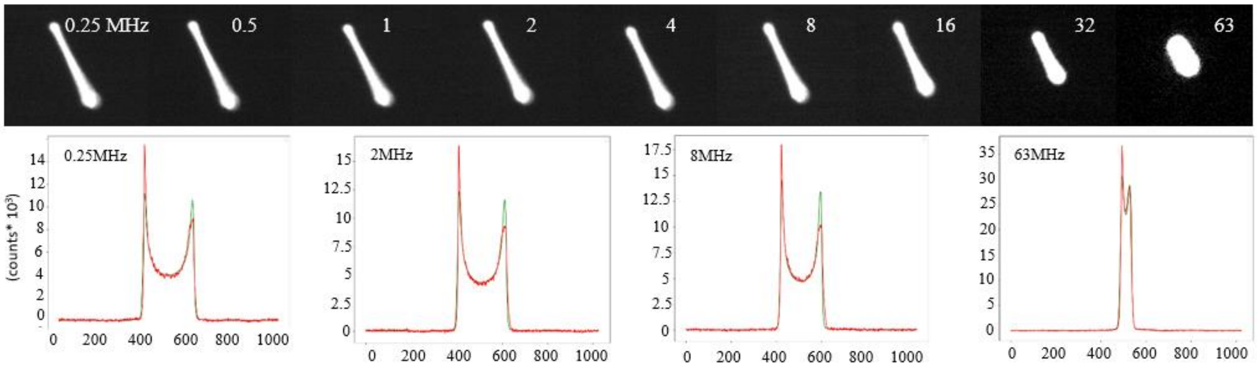

Supplementary Figure 5 | Anharmonic motion of electron beam at different frequencies. Anharmonic motion of electron beam in the far field at different frequencies. First row depicts whole beam and the red lines in the second row its projections on oscillator axis (i.e., *x*-axis corresponds to deflection angles in arbitrary units). The green lines are obtained from the slightly anharmonic oscillator model (Supplementary Note 6) fitting the free parameters of the model to the experimental (red) profiles.

Supplementary Figure 5 displays the integrated motion as a function of frequency. It is observed that the electron beam was oscillating in a slightly anharmonic fashion, in particular at small frequencies (large beam deflections). Additionally, the beam is smeared out at larger deflections. Both deviations from the ideal behavior may be attributed to either inhomogeneities in the field for larger excitations, or aberrations in the subsequent optics, induced by the tilt of the electron beam. The latter effect may

be more pronounced compared to a standard microscope setting, as the probe aberration corrector was used in an extraordinary setting to demagnify the beam from the C3 position with respect to the specimen position, in order to increase the corresponding deflection angles.

In order to analyze the deflection of the electron beam in a quantitative fashion we fitted the position distribution density $f(x)$ pertaining to a slightly anharmonic oscillator function to the intensity profile, which we assumed be a convolution of $f(x)$ with the probe function $g(x,y)$. The probe function was determined separately from a static beam exposure. The position distribution density of the slightly anharmonic oscillator may be derived from the motion of the anharmonic oscillator of basis frequency ω_0 and anharmonicity of strength ε

$$x(t) = \sin\omega_0 t + \frac{\varepsilon}{6\omega_0^2}(3 - 4\sin\omega_0 t - \cos^2\omega_0 t + \sin^2\omega_0 t) \tag{3}$$

Here, x was normalized such that the amplitude of the motion x_{m} is equal one. The position probability distribution may be found via the inverse function theorem (i.e., evaluating, how long the beam is at a certain position):

$$f(x) = \frac{1}{x'\big(t(x)\big)} = \frac{1}{\omega_0\cos\omega_0 t\,(x) + \frac{2\varepsilon}{3\omega_0}(-\cos\omega_0 t\,(x) + \cos\omega_0 t\sin\omega_0 t\,(x))} \tag{4}$$

Considering that the anharmonicity is small we may as a first approximation use the harmonic oscillator expression $t(x) = \sin^{-1} x$ to obtain

$$f(x) \approx \frac{1}{\omega_0\sqrt{1-x^2}\left(1 + \frac{2\varepsilon}{3\omega_0^2}(x-1)\right)} \tag{5}$$

We readily see that in the limit of vanishing ε the expression of the harmonic oscillator is recovered. The anharmonicity manifests itself as symmetry break with respect to the rest position. The whole fitting procedure of the intensities consisted of an initial normalization and slight denoising, followed by an alignment of the lines with respect to the x-axis. Then the intensity profile is projected along y (see line profiles Supplementary Figure 5) and finally fitted to the anharmonic oscillator model. In the line profiles pertaining to different frequencies, it can be observed that the fit worked very well. The obtained deflection magnitude exhibits a sigmoidal drop towards higher frequencies, starting at about 250 kHz. The minimal detected deflection above the noise level for this experiment was at 128 MHz, indicating the cut-off frequency of the current holder and device assembly.

Supplementary Note 7 | Deflection and Lissajous figures

A rich variety of curves can be formed by a superposition of two mutually perpendicular simple harmonic motions. If the ratio of the frequencies of the two motions is rational, the curves are closed and are referred to as Lissajous figures, which may be used in studying frequency, amplitude and phase relations of harmonic oscillators.

For harmonic motions with identical frequency and different phase, ellipses of varying angular positions and eccentricities are formed, which assume the shape of a perfect circle at a phase difference of 90°, whereas an "8" shape is achieved with a frequency ratio of 2:1 (see Fig. 4 f). The position-dependent blurring of the beam, observed in the patterns, can be attributed to deviations from the ideal superposition of two perpendicular homogeneous fields, generating an additional (de)focusing effect (see line profiles Supplementary Figure 6).

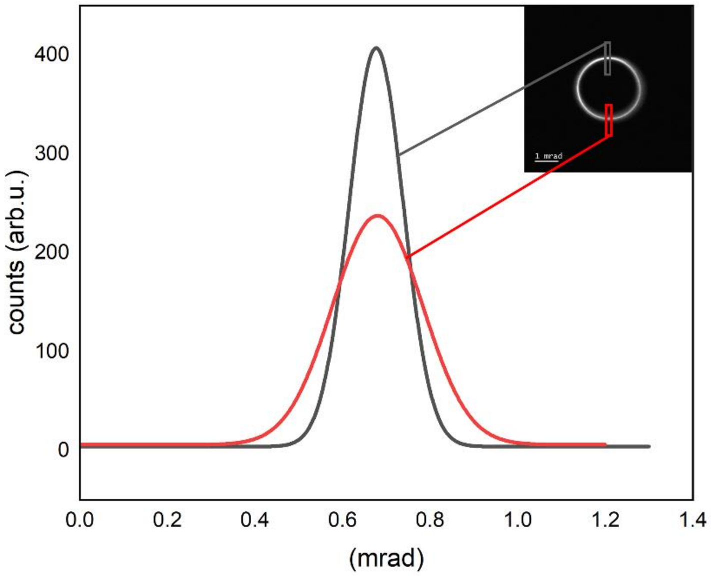

Supplementary Figure 6 | Cross section analysis of circular Lissajous pattern depicted in upper right corner. The two profiles taken at different positions of the Lissajous pattern contain approximately the same total electron intensity (area under the profiles), while differing in extension due to inhomogeneous field distribution and hence focusing.

Supplementary Note 8 | Alternative Magnetic Core Fabrication

In order to facilitate a complete parallelization of the fabrication process of the chips and improving the alignment of the magnetic poles and hence the parasitic aberrations, an alternative to the manual insertion of the soft magnetic core is required. Thus, preliminary experiments have been executed

to incorporate electroplating of the soft magnetic cores into the lithographic fabrication process as this approach would largely reduce misalignment of the poles and increase homogeneity and fabrication speed of the devices (see Supplementary Figure 7).

For the electroplating process, a copper layer of 400 nm is sputtered over the wafer, afterwards, a 50 µm AR-N 4450 (Allresist, Strausberg, Germany) photoresist is photopatterned on top of the copper layer, following the recommendations of the photoresist producer. During the electroplating step the patterned photoresist shapes the permalloy (Py) bars and the copper layer acts as an electrode. The employed electroplating solution is composed by 100 mL DI water with: 6g of Nickel(II)-sulfamat Tetrahydrat, 4g of $NiCl_2$, 0.8 g of $FeSO_4$, 3g of H3BO4, 5g of L-Ascorbic acid, 0.1g of Natriumdodecylsulfat, 3.1 g of Naphthalintrisulfonsäure Trinatriumsalz Hydrat Naphthalintrisulfonsäure Trinatriumsalz Hydrat, 0.08 g of 3-(1-Pyridin)-1-propansulfonat, and 0.5 g of Saccharine. All components are reagent plus grade and were ordered from Sigma Aldrich without further purification. The electroplating is performed with a DC current at 5 mA/cm^2.

Supplementary Figure 7 a and b show the schematics of a planar dipole after electroplating the Py bar and a wafer after electroplating the Py, respectively. While plating and shaping of the poles have been successful, we still struggle to reproduce the ultrasoft magnetic properties of the hand-inserted microwires (sustaining longitudinal vortex ground state), which requires further optimization of the material composition, the geometry of the filling and electroplating parameters.

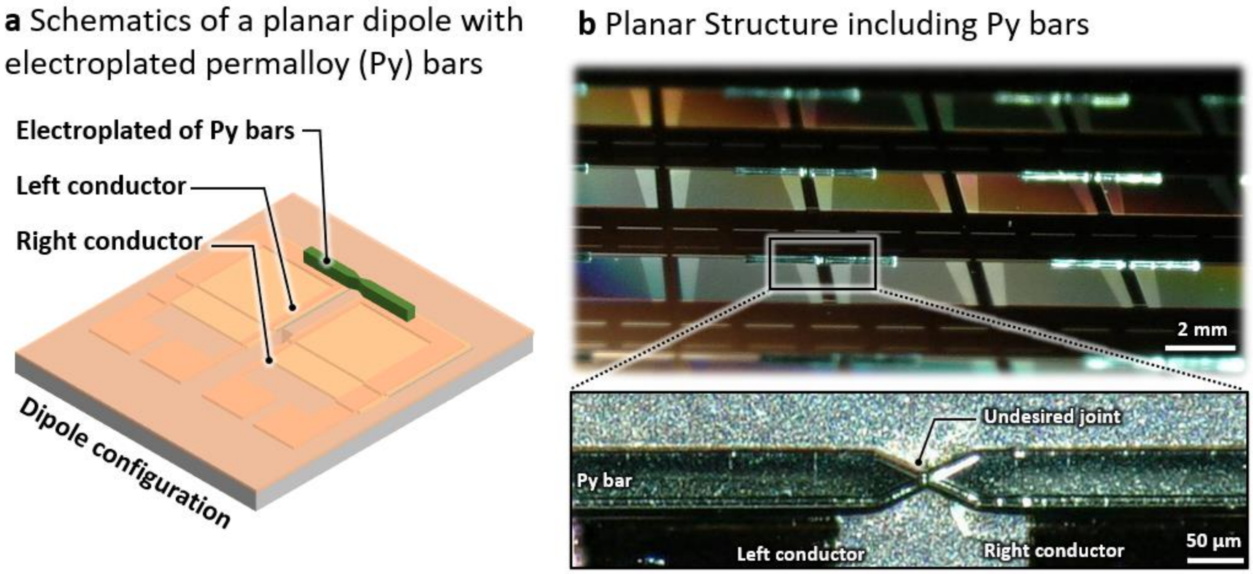

Supplementary Figure 7 | Soft-magnetic core electroplating. a, Schematic of the permalloy (Py) bar deposition on the planar chip design in the dipole configuration before self-assembly (roll up of the coils). **b,** Image of the wafer including Py bars with a zoom in of one Py bars over the aperture position. The undesired joint is opened in the next step by a short current pulse.

References

1. Karnaushenko, D. D. *et al.* Rolled-Up Self-Assembly of Compact Magnetic Inductors, Transformers, and Resonators. *Adv. Electron. Mater.* **4**, 1800298 (2018).
2. Becker, C. *et al.* Self-assembly of highly sensitive 3D magnetic field vector angular encoders. *Sci. Adv.* **5**, eaay7459 (2019).
3. Cendula, P., Kiravittaya, S., Mönch, I., Schumann, J. & Schmidt, O. G. Directional Roll-up of Nanomembranes Mediated by Wrinkling. *Nano Lett.* **11**, 236–240 (2011).
4. Karnaushenko, D. *et al.* Biomimetic Microelectronics for Regenerative Neuronal Cuff Implants. *Adv. Mater.* **27**, 6797–6805 (2015).
5. Béché, A., Van Boxem, R., Van Tendeloo, G. & Verbeeck, J. Magnetic monopole field exposed by electrons. *Nat. Phys.* **10**, 26–29 (2013).
6. Pozzi, G., Lu, P. H., Tavabi, A. H., Duchamp, M. & Dunin-Borkowski, R. E. Generation of electron vortex beams using line charges via the electrostatic Aharonov-Bohm effect. *Ultramicroscopy* **181**, 1339–1351 (2017).
